# Supplementary material for: Highly selective covalent organic functionalization of epitaxial graphene
Source: Nat Commun. 2017 May 8;8:15306. doi: 10.1038/ncomms15306 (PMC5424159; doi:10.1038/ncomms15306)
Supplement: Supplementary Information — Supplementary Figures, Supplementary Tables, Supplementary Notes, Supplementary Methods and Supplementary References [file ncomms15306-s1.pdf]

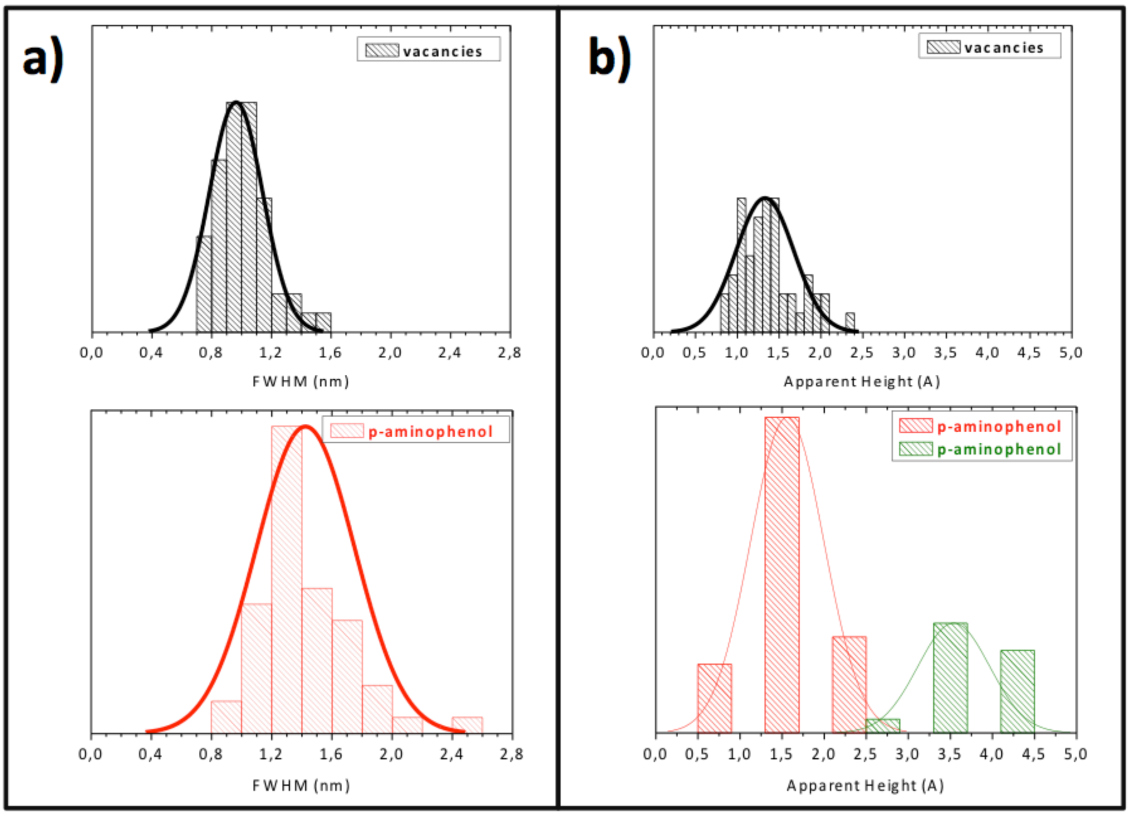

Supplementary Figure 1. FWHM and Apparent Height Histograms. Histograms of a) full width height medium (FWHM) and b) apparent height for vacancies and p-aminophenol.

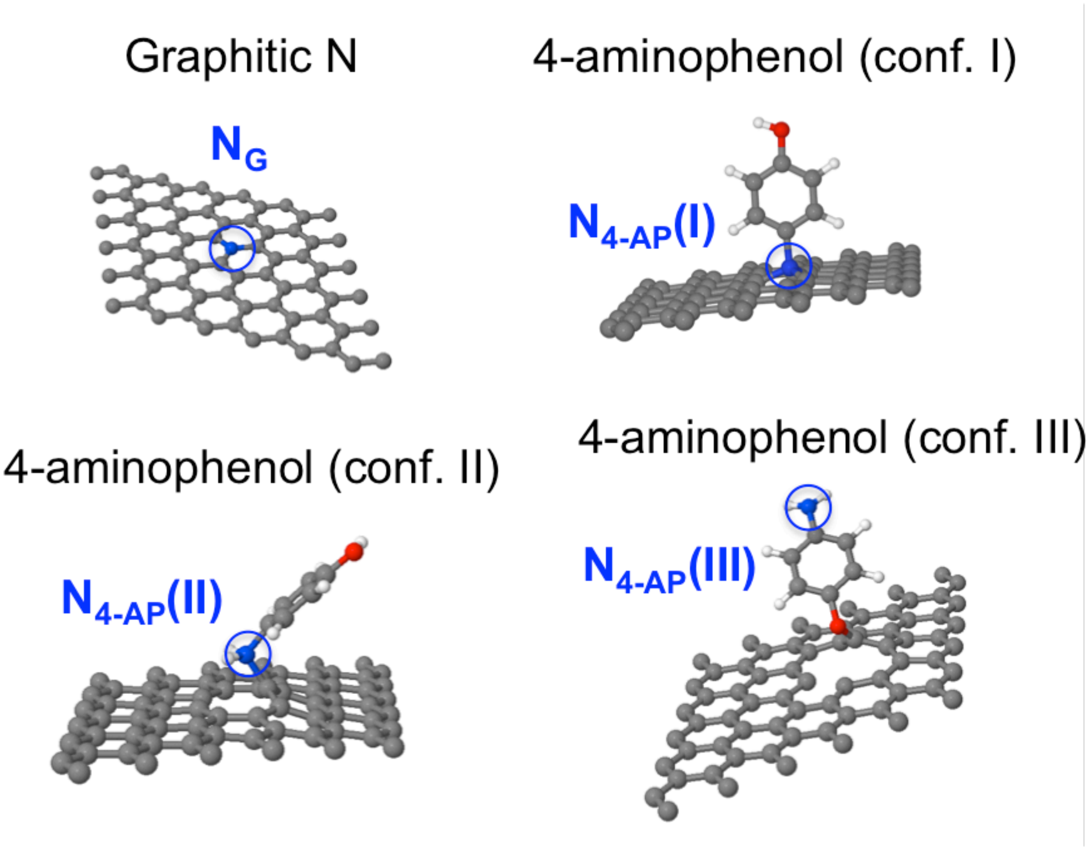

Supplementary Figure 2. Geometries used for the CLS Calculations. Optimized geometrical configurations used in our N 1s core level shift (CLS) calculations where the N atoms are in the following situations: (top-left) graphitic N, N_G, (top-right) doubly dehydrogenated N atom in the 4-AP molecule incorporated into the graphene lattice via a SAV, N4-AP(I), (bottom-left) N atom in the 4-AP molecule interacting with a SAV, N4-AP(II), (bottom-right) N atom in the 4-AP molecule with the dehydrogenated O atom interacting with a SAV N4-AP(III).

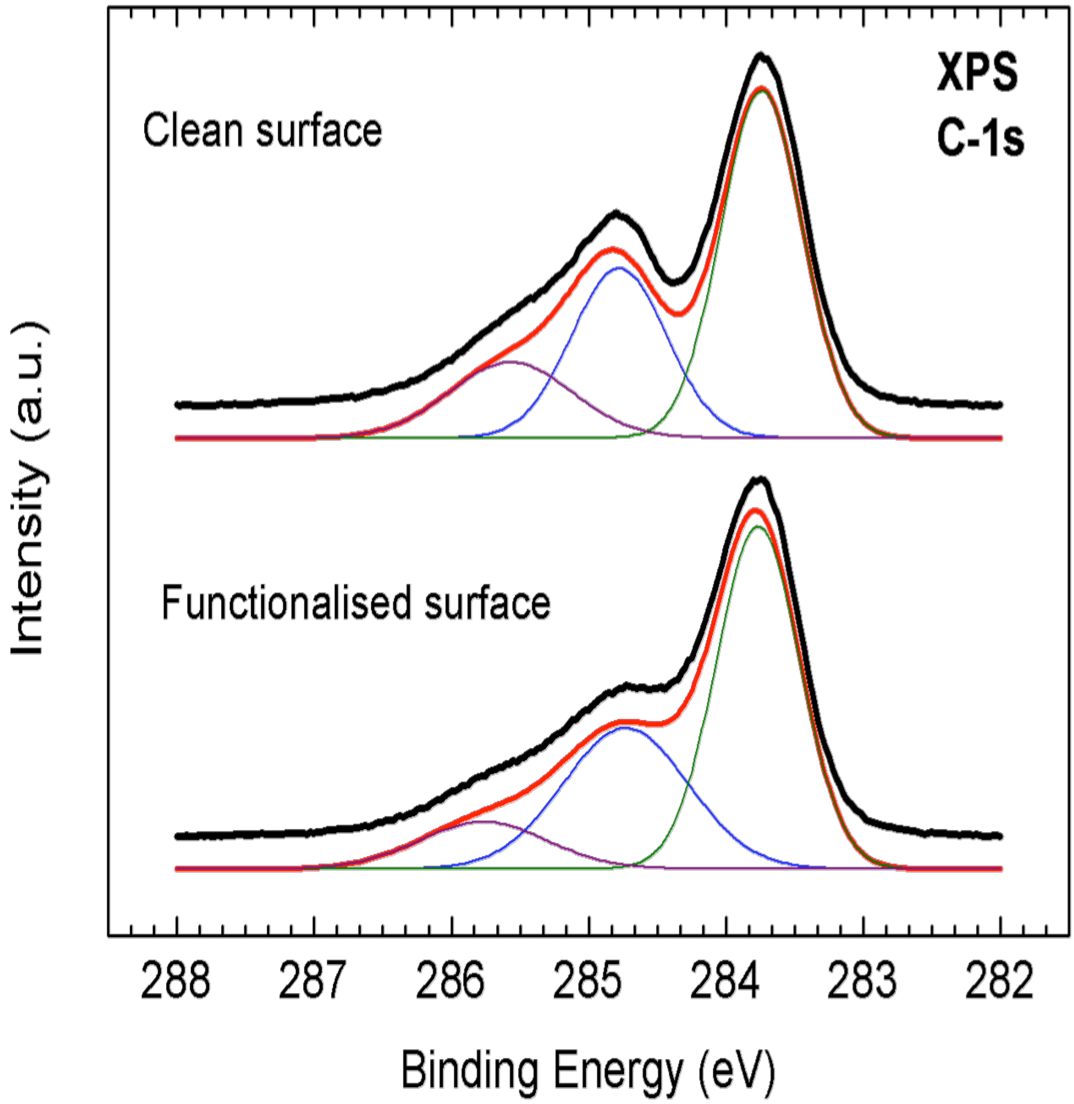

Supplementary Figure 3. Core-level Peak of C *1s* from the Clean and Functionalized Surface. The spectrum is fitted using three peaks associated to bulk SiC (green line), carbon sp^2 configuration (blue line) and SiC buffer (purple line) in increasing order of binding energy. The sp^2 area in functionalised surface increased respect to bulk SiC and SiC buffer areas due to the inclusion of the benzene ring of the p-aminophenol molecules.

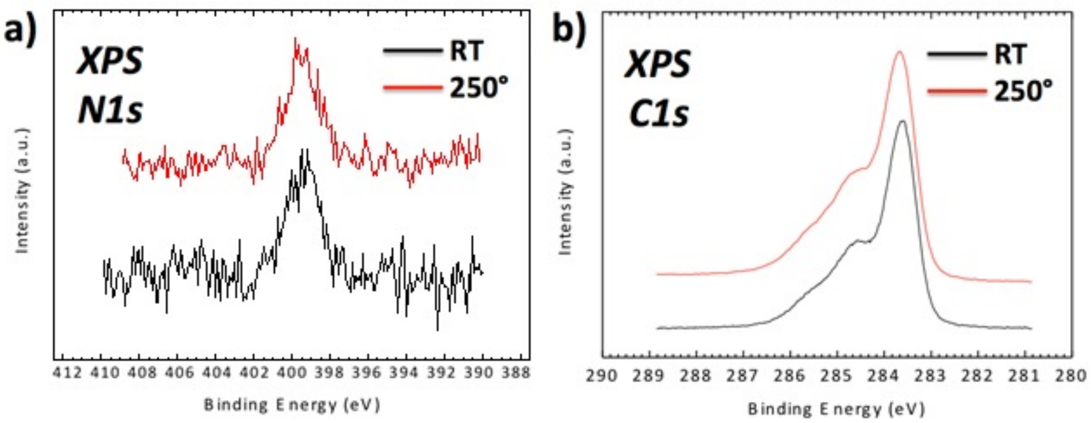

Supplementary Figure 4. XPS N *1s* and C *1s* Spectra at Different Temperature. a) N *1s* and b) C *1s* spectra of the functionalized surface of graphene on 4H-SiC(0001) before (black continuous line) and after (red continuous line) annealing to determine if bonds are covalently linked.

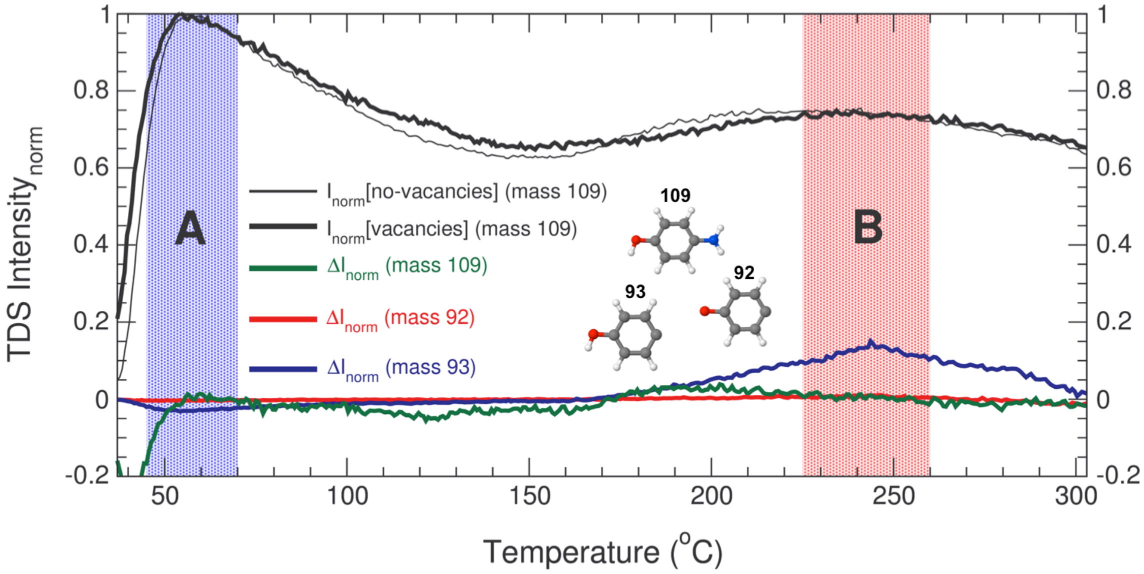

Supplementary Figure 5. Thermal Programmed Desorption Measurements. Normalized TPD Spectrum of a p-AP functionalized surface of SLG epitaxially grown on SiC(0001): (thin-black line) before SAVs creation, and (thick-black line) after SAVs creation. Blue-shaded zone A indicates the desorption of molecular adsorbed p-AP, taking place at the relatively low temperature of 333 K. Red-shaded zone B indicates a spurious signal stemming from p-AP molecules desorbing from the sample holder and sample manipulator. (Green line) Difference curve of the two p-AP TPD spectra in black for a mass of 109 amu. (Red and blue lines) Difference curves of two TPD spectra for two different p-AP fragments of masses 92 and 93 amu, respectively (fragments shown as inset).

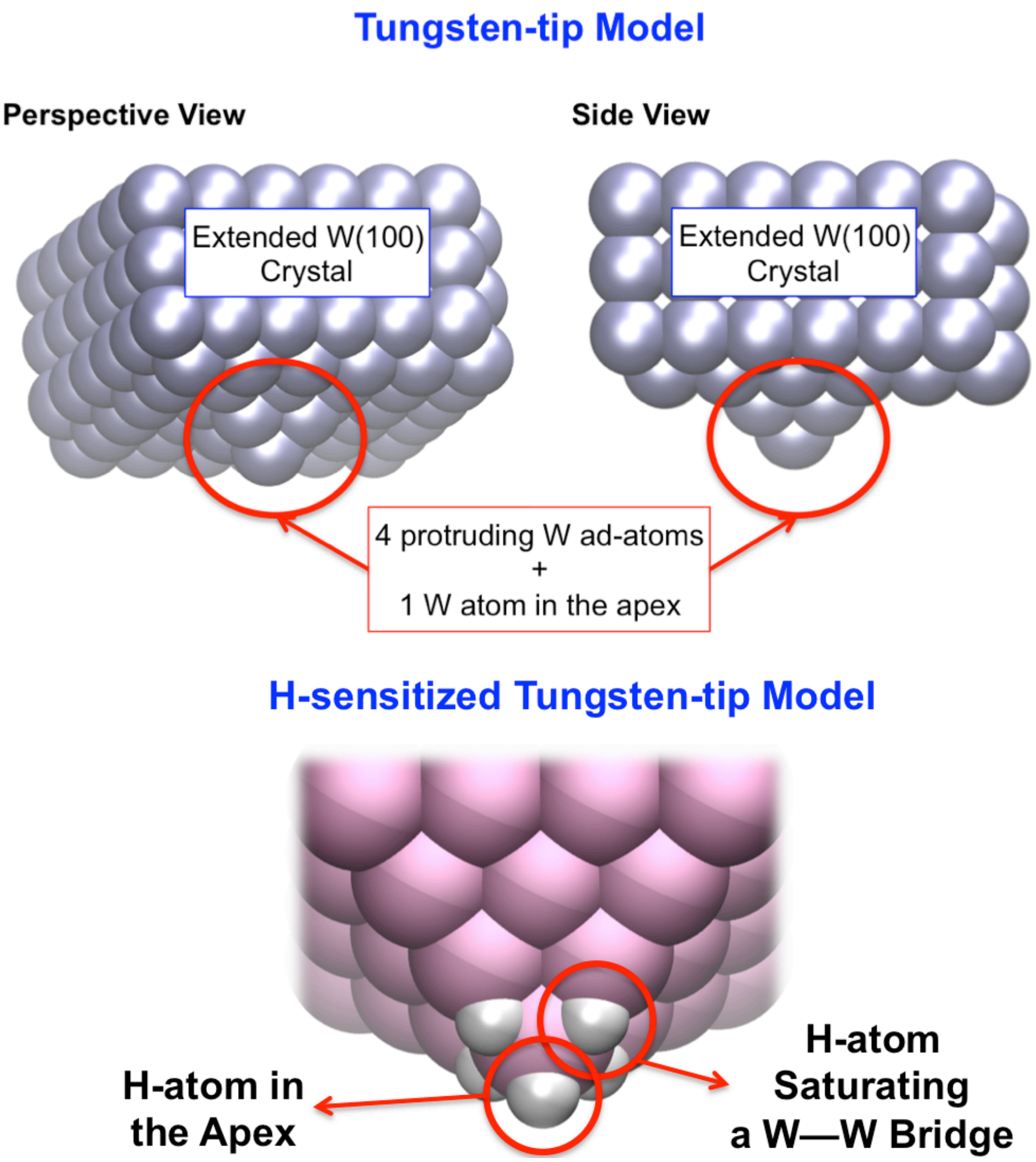

Supplementary Figure 6. W-tip and H-sensitized W-tip. Pictorial scheme of a clean tungsten-tip pyramidal model, and the H-sensitized tungsten-tip pyramidal model used in the calculations.

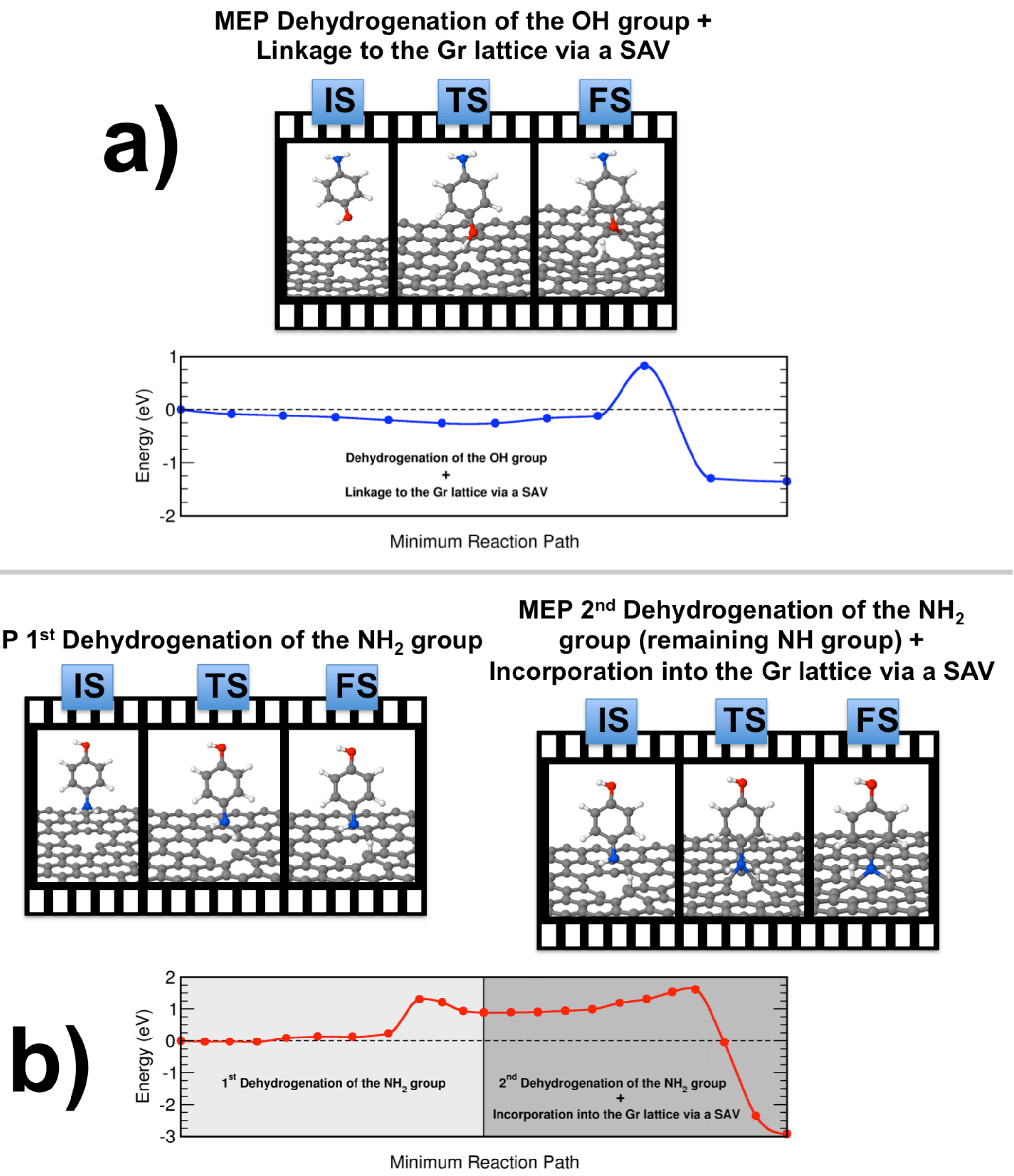

Supplementary Figure 7. Minimum Energy Paths. a) (Top panel) Most representative geometries (Initial State, IS, Transition State, TS, and Final State, FS) along the Minimum Energy Path (MEP) obtained within the CI-NEB approach for the dehydrogenation of the OH terminating group of the p-aminophenol molecule and the subsequent linkage of the dehydrogenated p-aminophenol molecule into the graphene lattice via a SAV from the O side. (Bottom panel) Energy (in eV) vs. reaction coordinate along MEP (referred to IS). b) (Top panel) Most representative geometries (IS, TS, and FS) MEP obtained within the CI-NEB approach for the first (left) dehydrogenation of the NH_2 terminating group of the p-aminophenol molecule; and (right) the second dehydrogenation of the NH_2 terminating group of the p-aminophenol molecule – first dehydrogenation of the remaining NH

terminating group – and the subsequent incorporation of the doubly dehydrogenated p-aminophenol molecule into the graphene lattice via a SAV from the N side. (Bottom panel) Energy (in eV) vs. reaction coordinate along MEP (referred to IS).

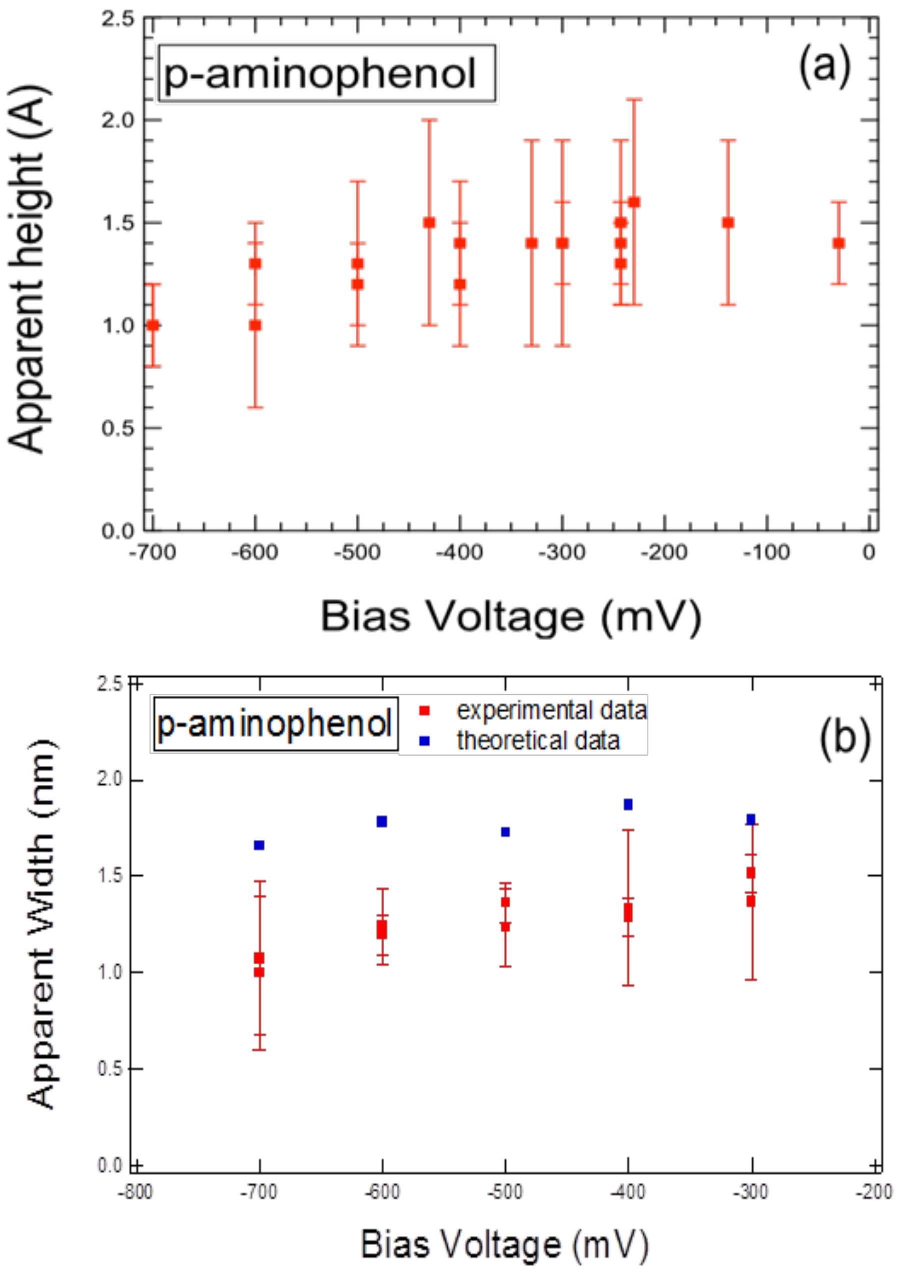

Supplementary Figure 8. Apparent Height and Width vs. Bias Voltage in STM Images. Apparent height (a) and Full Width at Half- Maxima (FWHM) (b) of the molecular features in the STM images as a function of the applied bias for the case of one p-aminophenol located at isolated mono-vacancies. The magnitude of the error bars has been taken as the difference between the maximum and minimum values extracted from our phenomenological statistics, for the same STM scanning area, centered at the corresponding average values.

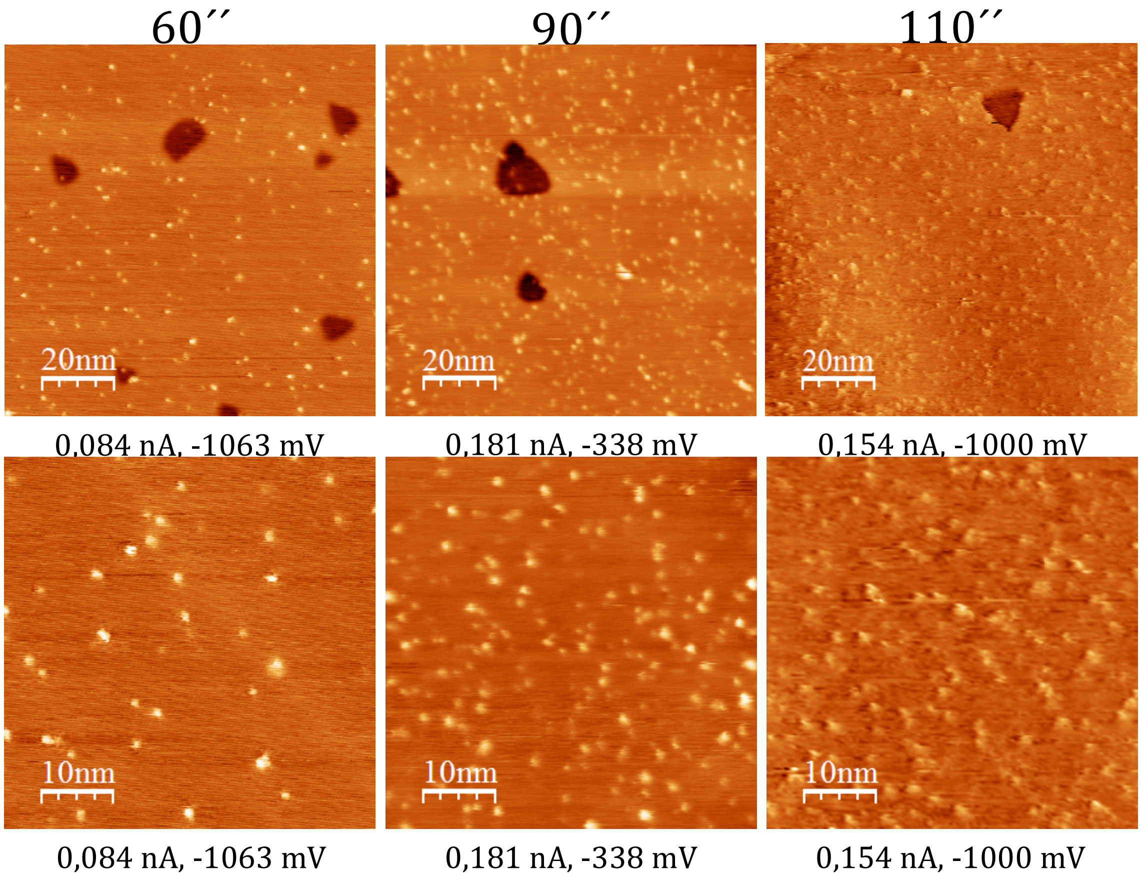

Supplementary Figure 9. STM Evolution of the Coverage with the Ion-dose. Images after p-AP Functionalization at Different Coverages. Some of the STM images used for counting the density of immobilized pAP molecules after Ar^+ irradiation with 60, 90 and 110 seconds, respectively. Upper row corresponds to a scanning area of 100×100 nm^2, and lower row to 50×50 nm^2 with the same ion-dose.

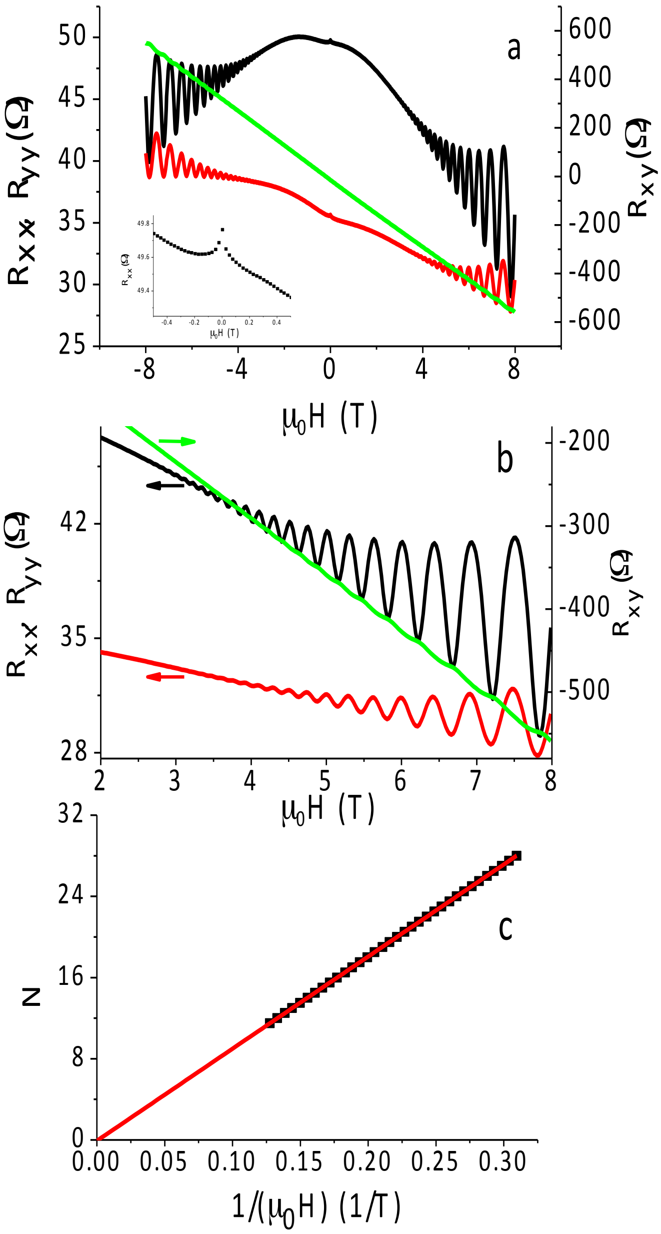

Supplementary Figure 10. Magnetotransport Measurements. (a) Resistance vs magnetic field for a p-AP implanted sample on an n-doped SiC substrate at 1.8 K, for the three measured channels along the x (black squares), y (red squares) and diagonal (green squares directions) directions. The inset shows the weak localisation peak near **H**=0 Oe for the R_{xx} channel. (b) Shubnikov-de Haas oscillations in the raw data for positive magnetic field at 1.8 K. Same colour code as in (a). (c) Linear dependence of Landau level index, *N*, with inverse magnetic field. Integer (half-integer) values of *N* have been assigned to oscillation maxima (minima) in channel R_{xx} after subtraction of a cubic polynomial from raw resistance data.

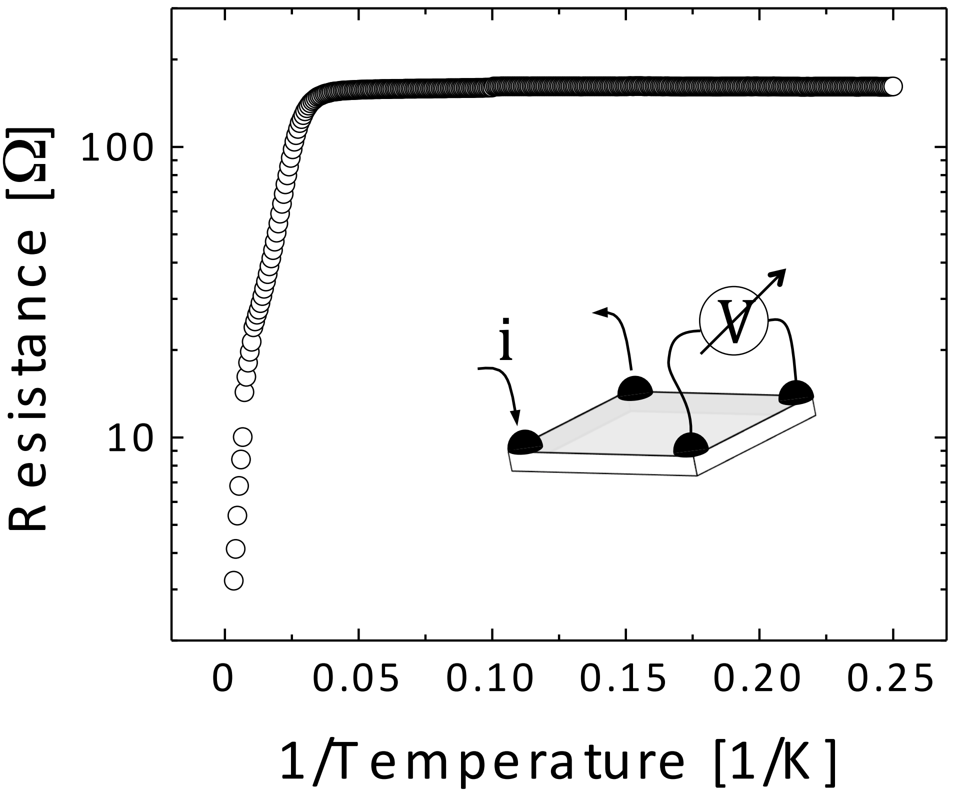

Supplementary Figure 11. *R* vs. 1/*T*. Resistance vs. inverse temperature at zero magnetic field for the p-AP functionalized sample on a semi-insulator SiC substrate. Inset shows the 4-wire van der Pauw configuration used for the electrical contacts.

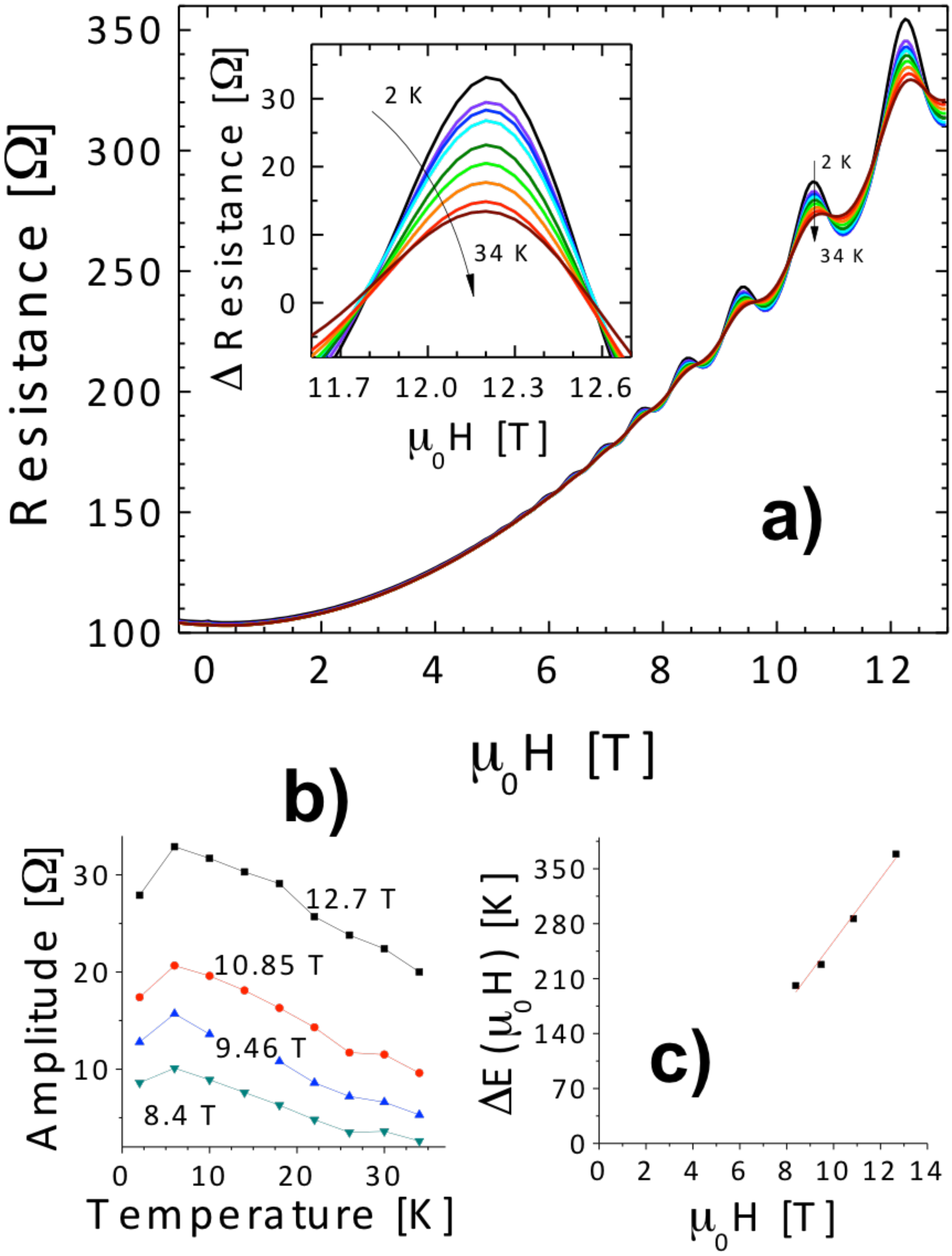

Supplementary Figure 12. Shubnikov-de Haas Oscillations. (a) Shubnikov-de Haas oscillations in the R_{xx} channel for a sample on a semi-insulator SiC substrate with $m_0\mathbf{H}_0$ = (75.4 ± 0.1) T and g = (0.02 ± 0.2). (b) Amplitude of the oscillations at the higher magnetic fields measured. (c) Field dependence of the separation between of the Landau levels as function of magnetic field.

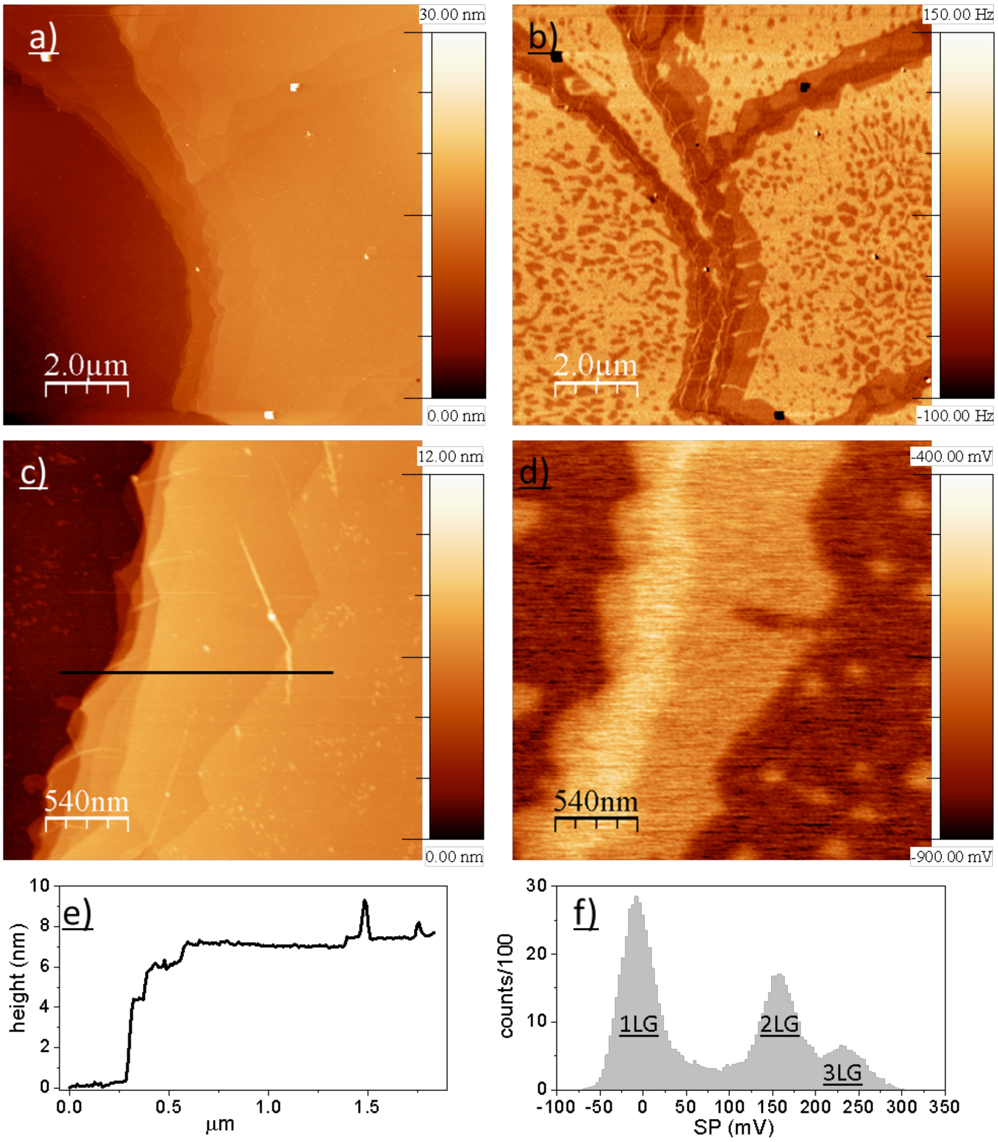

Supplementary Figure 13. Morphological and Electrostatic Characterization after p-AP Functionalization. Simultaneous a) topography and b) frequency shift AFM images of the epitaxially grown graphene on SiC(0001) after p-AP functionalization (10×10) µm^2. Simultaneous c) topographic and d) surface potential KPM images of the p-AP functionalized graphene (2.7×2.7) µm^2. e) Height profile across a step bunch along the corresponding line in c). f) Histogram corresponding to the surface potential map in d), illustrating the relative electrostatic difference values between one (1LG), two (2LG) and three (3LG) graphene layers.

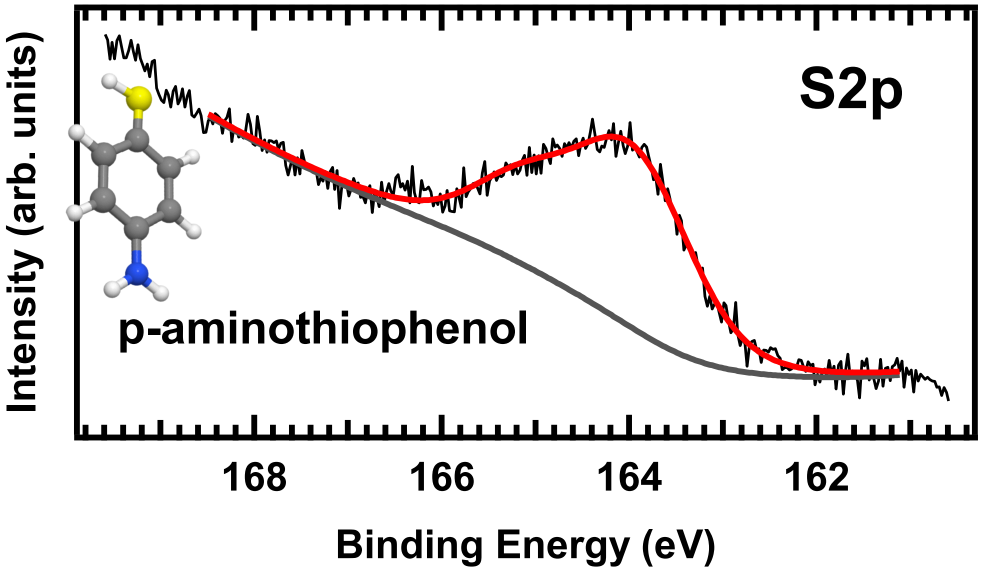

Supplementary Figure 14. XPS S *2p* Core-level of Functionalized 4H-SiC(0001) with p-aminothiophenol Molecules (Same Procedure that for p-AP). The fit is achieved with a single peak indicating unbound thiol group.

Supplementary Table 1. N *1s* CLS Calculations. Results for the core level shift of the N *1s* in the different configurations shown in Supplementary Figure 2 w.r.t. a graphitic N atom, N_G, obtained from our calculations.

Atom	$Atom^{ref}$	CLS_{final}(eV) – TM -	CLS_{final}(eV) – RRKJ -
N_{4-AP}(I)	N_G	+1.23	+1.31
N_{4-AP}(II)	N_G	+0.67	+0.76
N_{4-AP}(III)	N_G	-0.25	-0.34

Supplementary Table 2. N *1s* Calculated Binding Energies. Results for the binding energies of the N *1s* in the different configurations shown in Supplementary Figure 2, taking graphitic N binding energy to be 400.5 eV,[1] obtained from our calculations.

Atom	$Atom^{ref}$	Binding energy (eV) – TM -	Binding energy (eV) – RRKJ -
N_{4-AP}(I)	N_G	399.27	399.19
N_{4-AP}(II)	N_G	399.83	399.74
N_{4-AP}(III)	N_G	400.75	400.84

Supplementary Table 3. Previous XPS-related Literature. Previous literature of related species to position our binding energy with respect to somehow similar chemical groups.

**Chemical Species**	**Graphitic N**	**R-N-C**	**$-NH_2$**	**-N=**	**-NH-**	**$R-NH_2$ Cysteine**	**Our experiment**
**References**	*1, 2, 3*	*4*	*5*	*5*	*5, 6*	*7*	
**Binding Energy (eV)**	*400.5, 400.6, 400.9*	*399.7*	*401.1*	*398.9*	*400.1*	*401.3*	*399.5*

Supplementary Note 1: Modellization of Systems for the Computational Analysis

In order to design an accurate and trustable structural workbench for constructing the different geometrical models that we have used in the calculations (towards the monovacancy creation and the subsequent integration into them of p-AP molecules) we have taken advantage of a 1650 atom supercell considered in previous literature by our group.[8] This model consists of four (6√3×6√3)R30° SiC bilayers terminated by hydrogen to maintain the charge balance (see Figure 1). The cell was capped by a honeycomb carbon mesh that strongly buckles upon relaxation, the buffer layer. On top of it, we placed a commensurated SLG. All the atoms were allowed to relax except for the last SiC bilayer and hydrogen layers. The stacking of the SiC bilayers corresponded to the 4H-SiC polymorph. The lattice parameter of the SiC unit cell was obtained from previous works.[9] The relaxed structure of this unit cell can be described as follows: four slabs of SiC pairs presenting an atomic configuration relatively close to the hexagonal SiC bulk configuration, Si−C bond distance of 1.9 Å and SiC−SiC interlayer distance of 2.6 Å, compared with experimental 1.89 and 2.52 Å.[8] The SiC slabs are capped by the buffer layer, a highly buckled honeycomb carbon mesh with a peak-to-peak corrugation of around 1.1 Å. This strong corrugation within the honeycomb carbon lattice of the buffer layer breaks the sp^2 hybridization and induces the bonding between some of the carbon atoms within this buffer layer and some of the Si atoms of the last unperturbed SiC slab underneath. Therefore, the properties of the buffer layer are far from those of pristine graphene, although they both share the honeycomb carbon lattice as its basic structure. Finally, on top of all, at an average vertical distance of 3.7 Å over the buffer layer, we find a pure (13×13) sheet of SLG with a negligible corrugation below 0.1 Å. Nevertheless, to make the computationally demanding present calculations more manageable we have removed the SiC substrate and left the SiC buffer layer, saturating the broken bonds arising from C atoms with hydrogen atoms. In all the structural relaxations this buffer layer has been kept fixed. This assumption turns into quite acceptable with a high degree of confidence in the sense that following this strategy the SLG is

highly decoupled from the buffer layer, which is reinforced by the experimental observations and the high-quality experimental STM images obtained for the graphene.

Supplementary Note 2: Statistical Counting of Covalently Linked p-AP Molecules and Vacancies

The determination of the number of vacancies/p-aminophenol was performed over 12 STM images of size (100×100) nm^2 recorded on different surface regions to improve the statistical analysis. The results have revealed that the average concentration of p-aminophenol covalently linked to graphene at the vacancy sites is 3.48 $\times 10^{-3}$ molecules per C-atom.

Supplementary Figure 1a shows a histogram of full width height medium (FWHM) of 50 vacancies (top panel; black) and 50 p-aminophenol molecules (bottom panel; red) extracted from a large variety of STM images, which were acquired in different zones of the sample and at several bias and current conditions.

In top panel of Supplementary Figure 1a we observe that the FWHM corresponding to isolated mono-vacancies (with sufficient lateral separation between them) is well defined and exhibits a sharp shape with a maximum located at around 1 nm. Nevertheless, after the incorporation of the p-aminophenol the curve fitting is significantly shifted to higher values, up to 1.4 nm in average due to p-aminophenol contribution to the graphene lattice. In this case the range of values turns into slightly wider, enlarging the shape of the histogram towards higher values. This range of values may be explained in terms that if the mono-vacancies are very close from each other, and the molecules are added, the p-aminophenol electronic contribution is underhanded in STM images; as a result, wider and higher bumps and a less inhomogeneous appearance are shown in STM images in comparison with mono-vacancies STM images.

In the same way, histograms of apparent heights are presented in Supplementary Figure 2b. Here, the vacancies curve (top panel; black) is quite narrow with a maximum located in average at 1.2 Å and the molecules curve (bottom panel; red)

is substantially more affected by the electronic p-aminophenol contribution, hereby a much wider curve with a maximum located at 1.6 Å. In the functionalized images of Supplementary Figure 2b, the green values correspond to features that overlap to each other, and therefore shall not be taken into account.

At this point it is important to notice the effect of bias in width and height, which cause the previously presented distribution to become wider. The variation of these parameters vs. the applied bias is represented in Supplementary Figure 8 for the case of one p-aminophenol molecule located at an isolated mono-vacancy, away from other molecule contributions.

In left panel of Supplementary Figure 8, when the bias voltage increases from -300 to -700 mV the width decreases from 1.5 to 1 nm with a certain scalable trend. However, in right panel of Supplementary Figure 8 there is not a visible clear tendency and no significant changes are observed, yielding values within a range between 1 and 1.5 Å, which manifests that the apparent height seems to be independent of the applied bias up to -700 mV. This non-scalable behaviour for the apparent height as a function of the applied bias is also concluded from regarding theoretical STM scanning lines along fix trajectories, obtaining values ranging randomly between 1.7 and 1.9 Å, varying the applied bias from -700 mV to -300 mV. These results are coincident with most of the statistics of the histograms in Supplementary Figure 1 and STM images, which we can conclude that is the most common case. Note that right-hand Supplementary Figure 8 has been made using both, functionalized and non-functionalized images.

Supplementary Note 3: Exploring N *1s* Core-level-shift by DFT-based Calculations

In order to rationalize the origin of the N *1s* core-level-shift observed in our XPS experiments we have carried out a set of DFT-based calculations with the plane-wave code QUANTUM ESPRESSO[10] for different molecule adsorption configurations. For the calculations of core level binding energy shifts we have employed the final state approximation,[11-13] where all the contributions are

accounted (initial approximation + difference in screening response between the original and the reference systems). First principles calculations within this approach have been successfully applied in many different studies ranging from core level shifts in small molecules, nanowires and clusters[13-15] to surface core level shifts.[16,17] Details of the calculations are the following: i) fully-relaxed geomtries, ii) calculations with hard Troullier-Martins (TM)[18] and ultrasoft Rabe-Rappe-Kaxiras-Johanopoulos (RRKJ),[19] iii) a dense grid of (16×16×1) k-points to obtain converged results up to 0.01 eV in total energy differences, and iv) final state-approximation (FSA), where valence charge has been permitted to fully self-consistently relax and converge.

Since we are using pseudopotentials, only binding energy differences (shifts) for core states on a given atom are relevant for a comparison with experimental XPS observations. Then, taking a given atom as reference (in our case the N atom in a purely graphitic configuration; see top-left panel in Supplementary Figure 2), the chemical shift for the atom of our interest w.r.t. the reference will be just the difference between the computed core level energies for the N atom in the different molecular adsorption configurations (see Supplementary Figure 2) w.r.t. the reference atom, N_G, considered as follows:

$$CLS_{final}[N_{4-AP}(I)] = [E_{1s*hole}(N_{4-AP}(I)) - E_{ground}(N_{4-AP}(I))] - [E_{1s*hole}(N_G) - E_{ground}(N_G)]^{ref}$$
$$CLS_{final}[N_{4-AP}(II)] = [E_{1s*hole}(N_{4-AP}(II)) - E_{ground}(N_{4-AP}(II))] - [E_{1s*hole}(N_G) - E_{ground}(N_G)]^{ref}$$
$$CLS_{final}[N_{4-AP}(III)] = [E_{1s*hole}(N_{4-AP}(III)) - E_{ground}(N_{4-AP}(III))] - [E_{1s*hole}(N_G) - E_{ground}(N_G)]^{ref}$$

Results for the core level shift of the N *1s* in the different configurations (shown in Supplementary Figure 2) w.r.t. a graphitic N atom, N_G, obtained from our calculations are summarized in the Supplementary Table 1 (values obtained by using both TM and RRKJ pseudopotentials). And taking graphitic N binding energy to be 400.5 eV,[1] the binding energies obtained from our calculations would be those shown in Supplementary Table 2. According to the binding energy results showed in Supplementary Table 2, the best agreement with our N *1s* XPS experiment (399.5 eV) is obtained for the model we propose in the present study (399.27 eV), in which the 4-AP molecule incorporates into the graphene lattice via a SAV through its doubly dehydrogenated N atom (see Supplementary Figure 2).

Supplementary Note 4: Line-shape and Intensity Analysis of the C *1s*

Supplementary Figure 3 shows the C *1s* core level peak before and after functionalization. This peak is quite complex, as it contains components about the called buffer layer of SiC and bulk SiC. The fit using three components is quite standard in the literature.[20] The component at 285.57 eV can be assigned to bulk SiC, whereas the component at 284.78 to the C from the buffer layer. Finally the component at 283.74 corresponds to C sp^2.

The component of sp^2 shall increase its intensity after functionalization with respect to the other components, as the C in the aminophenol ring is sp^2 and the process does not affect the bulk components. This is indeed what happens. From the figure we measured the sp^2 area normalized to the C from buffer and bulk SiC and we obtained values of 0.43 and 0.51, for clean and functionalized surface, respectively. This variation is about 20%, number that may seem large with respect to our low coverage, but one has to take into account that we estimate a ratio rather than an absolute value. This number considers both the increase of the signal due to the adsorbed molecules and also the electron attenuation for the signal of the bulk component.

Supplementary Note 5: Binding Energy References

To assign core-level XPS binding energies to molecular species usually references to previously published works are used. However, in our case, the molecular adsorption we claim has not been previously reported. In order to find a valid reference we have looked for existing previous literature of related species to position our binding energy with respect to somehow similar chemical groups. The Supplementary Table 3 summarizes these values.

We have experimentally checked that we do not have N_2 adsorption, or even physisorbed molecules on the graphene surface (see spectrum in Figure 1g), in which we dosed aminophenol on pristine graphene – without any vacancy – and we were not able to see any N *1s* core level peak nor any trace on the STM (see Figure 1g). Therefore, after the formation of the vacancies, any remaining species must be covalently bound, as they are able to support high temperature without alteration. We can foresee only 3 possible scenarios:

Purely graphitic N. This would be the case if the molecule breaks upon reaction with the vacancy. This case has been deeply studied recently and there exist a bunch of references. We can take as a good reference the ref. (1) where, for sure, they have implanted N within the graphene lattice, and the binding energy for this system would be 400.5 eV. More than 1 eV shifted up with respect to our peak. Statistically, we have found that this value is quite accurate; and using other references we found a dispersion lower than 0.5 eV. Therefore, we can discard that the molecule is broken in our experiment, and that our N is substitutional.

Molecular adsorption by phenol group. In this case the amino group will stand out of the molecule, far from the surface, and it would lead to a configuration that is not very stable by energy arguments, since the oxygen atom is not able to fully saturate electronically the open vacancy (see Theoretical Section in Supplementary Methods, Supplementary Figure 7 and Supplementary Notes 8 and 9). In this case, we can expect that the binding energy will be, somehow, similar to that obtained on chemisorbed molecules that expose an amino group, as $–NH_2$ species. As an example, the cysteine molecule, which bound by the S to the gold surface, appears at 401.3 eV, value far away from our experimental observation or the $–NH_2$ group in biomolecules has been reported at an energy value of 400.1 eV. Therefore, we can discard this possibility.

Amino bonding to the surface due to a molecular cracking. Similarly to the previous case, the reported binding energy for this scenario is 400.1 eV (see Supplementary Table 3). This value is far from our experimental XPS observation.

Thus, from the Supplementary Table 3, summarizing previous related literature, it is evident that the closest value is the labelled as R-N-C, which can make us to exclude graphitic N, $-NH_2$ and $R-NH_2$.

Supplementary Note 6: Thermal Stability

To obtain information about the stability of the bond between the graphene network and the molecules at the vacancy sites, as well as to evaluate the degradation of the p-aminophenol by thermal treatments, we performed X-ray photoelectron spectroscopy (XPS) before and after heating at 250°C. The XPS measurements were performed in a UHV chamber, which base pressure of 2×10^{-10} mbar.

The p-aminophenol molecules are formed by an aromatic ring with an amino group and a hydroxyl group. Then, the most relevant elements for their study are the nitrogen (N *1s*) and the carbon (C *1s*) levels. Supplementary Figure 4 shows the spectra of these levels before and after the heat treatment, which consists on annealing the functionalized graphene/4H-SiC(0001) substrate at 250°C during 15 minutes.

In both cases, the N *1s* and C *1s* XPS curves reveal that the spectral shape, before and after annealing, do not experience any substantial change. This result confirms the thermal stability of the bonds between molecules and graphene network up to 250°C. Additionally, it evidences that the molecules do not undergo degradation processes up to 250°C, indicating the covalent bonding of the layer. This temperature is very conservative and has to be regarded as a minimum value, limited by our experimental capabilities.

In TPD experiments we do not have seen any signal coming from the molecule and therefore we can conclude that in this range of temperatures there is no desorption or rupture of the linked molecule. A summary of the TPD experiments can be found in Supplementary Note 7. This is what one may expect from a covalently linked molecule. Similarly, we have repeated the same experiment at

the STM, and neither modification of the molecular related features nor desorption were observed up 300°C.

Supplementary Note 7: Thermal Programmed Desorption

To get further insight into the thermal stability of the molecules functionalizing the graphene lattice we have carried out Thermal Programmed Desorption (TPD) experiments in a UHV chamber with a base pressure of 2×10^{-10} mbar. The samples were mounted unto the standard Mo Omicron sample flags, compatible with our home-build sample stage. The temperature ramps were controlled with an in-house developed LabVIEW program that has an interface to synchronize with the Pfeiffer h6T software, and samples were heated by using a pyrolytic graphite / pyrolytic boron nitride composite heater from Ceramisis. Temperature was measured using a k-type thermocouple, and was verified with pyrometer. Mass spectra were acquired with a Pfeiffer HiQuad QMG 700 with QMA 400 mass spectrometer (mass range of 0 to 512 amu; resolution of 0.3 amu as 10% of peak height), and a CP 400 ion counter preamplifier (detection limit of 10^{-15} mbar). For TPD experiments the sample was placed near (< 1 cm) the ionizer of the mass spectrometer. Due to the absence of a Feulner cup molecules desorbing from the sample holder and sample manipulator contribute significantly to the signal.[21] The degas procedure was kept the same as for the STM samples. The SAVs were created in situ using the same model of electron gun, under the same experimental conditions.

In Supplementary Figure 5 we show the recorded TPD spectra of p-AP desorbing from a surface of SLG epitaxially grown on SiC(0001) before and after creating SAVs. Also shown are the difference spectra from TPD signals before and after SAV creation coming from p-AP (with a mass of 109 amu) and two of its fragments (with 92 and 93 amu). Two features labelled with A and B can be observed in the TPD spectra. Feature A is the most intense and best defined of the two. Due to: i) its higher intensity, ii) its recognisable line shape of a first order desorption

process, and iii) an absence of significant increase of the total pressure in the vacuum chamber, we can assign blue-shaded zone A in the Figure to p-AP molecules originating from a homogeneous source close to the mass spectrometer ionization source i.e., the sample surface. The difference spectra show virtually no signal in the range of feature A, so we conclude that the p-AP molecules that are not bonded to a SAV desorb in the same way, independently of the presence of the SAV. The peak temperature of 60°C coincides well with STM experiments, where it was observed that a small annealing up to 100°C – or waiting for several hours at RT – improved significantly the stability of the measurements after depositing p-AP.

On the other hand, feature B (red-shaded zone in the Figure) exhibits a very broad ill-defined and less intense structure, and can be attributed to background signal stemming from molecules reaching the spectrometer indirectly and from inhomogeneous sources i.e., originating from sample holder and sample manipulator. This observation also coincides with a noticeable increase of the total pressure in the vacuum chamber due to molecules desorbing from the various surfaces. This background signal is expected due to the aforementioned absence of a Feulner cup. A small difference can be observed in the difference spectra within the range of zone B. As the sample has been moved in between the two TPD runs, some small changes in the background are to be expected and are within the uncertainties of our TPD setup.

Supplementary Methods

Experimental Section

General information

Epitaxial graphene was grown on the 4H-SiC Si-face samples[22] by chemical vapour deposition (CVD) at 1600°C using propane gas as the carbon source under an argon (Ar) laminar flow in a hot-wall Aixtron VP508 reactor. This procedure

yielded square-shaped samples of size (10×10×0.5) mm^3 of epitaxial graphene on 4H-SiC(0001) exhibiting a slight n-type doping character. In order to avoid any possible metal contamination the sample was taken out from the reactor by plastic tweezers and mounted in a tantalum sample holder to be finally introduced inside the ultra-high vacuum (UHV) chamber.

Experiments were carried out in a UHV chamber with a base pressure of 1×10^{-10} mbar, equipped with an OMICRON low energy electron diffraction (LEED) optics and OMICRON RT scanning tunnelling microscope (STM). STM images were acquired using topographic and current modes with bias values ranging from -700 to -300 mV and currents ranging between 0.01 and 0.1 nA. WSxM software was used for data acquisition and image analysis.[23] As observed in the STM images, the resulting surface was mostly formed by graphene monolayer (ML) regions; nevertheless, graphene buffer layer (BFL), bilayer (BL) and trilayer (TL) regions were also present.

Samples were degassed by annealing at 250°C during 15 minutes in order to remove the physisorbed contamination. This thermal treatment was carried out by electron irradiation in a heating stage placed at the manipulator, while the annealing temperature was carefully monitored with an infrared pyrometer (emissivity of 0.9). The single atom vacancies (SAV) were created in the graphene lattice by sputtering with Argon ions (Ar^+) during 45 seconds at a 0° angle incidence. The acceleration energy of the electrons inside the Specs IQE 11/35 ion gun was 140 eV, the current sample was 1μA and the Ar pressure was kept at 1×10^{-7} mbar.

Before dosing p-aminophenol molecules (Sigma Aldrich, purity: 99%), they were purified by several turbo-pumping cycles. There was no need to anneal the p-AP since it evaporates at RT. After that, we evaporated 10 Langmuir of p-aminophenol (1L = 10 s at 10^{-7} Torr) keeping the substrate at RT. Following this strategy we ensure that the organic molecules filled most of the SAV defects.

The X-ray photoelectron spectroscopy XPS measurements were taken in a UHV chamber with base pressure of 1×10^{-10} mbar using a DLD_Phoibos H5a3500

analyser with a monochromatic Aluminium source (energy of photons hν=1486.74 eV), with sensitivity deeper (bulk) than Mg source.

ARPES spectra have been acquired at RT at BadElph beamline located at Elettra Synchroton in Trieste using a SPECS Phoibos 150 with a 2D-CCD detector system. The energy resolution of the beamline is 4 meV, with an angular resolution of 0.1°. All the spectra were acquired twice, with vertical and horizontal polarization at hν=34 eV. No radiation damage of the sample was observed.

Electric transport and magneto-transport was investigated over macroscopic dimensions (10×10) nm^2 as a function of temperature (between room temperature and 1.8 K) and magnetic field (**H**) perpendicular to the graphene plane using two Quantum Design ® PPMS systems capable of reaching 9 and 14 T respectively. Samples on n-type or semi-insulator substrates were used as grown or after p-aminophenol (p-AP) implantation, without any further lithography processing and under a 4-5 Torr He atmosphere. Samples were initially kept during a four-hour period at room temperature in the PPMS sample chamber at the operation pressure. Contacts were provided in square van der Pauw configuration using Wimbush ® press-on devices over Cu-foil pads. Resistance measurements were collected *in-plane* in three measurement channels: along the *x*, *y* and diagonal directions (labelled *xx*, *yy* and *xy*, respectively). The first two channels are, therefore, optimised for resistivity measurements, while R_{xy} should provide a good approximation to Hall resistances.

Morphological characterization of the functionalized samples was performed by atomic force microscopy (AFM). Kelvin Probe Microscopy (KPM) imaging was additionally applied to simultaneously obtain an electrostatic map of the surface. This latter technique has been successfully used to the study of graphene, giving essential information on graphene thickness, layer-dependent distribution of charge, electrical potential and work function.[24] A commercial system from Nanotec, operating in ambient conditions, was employed. Measurements were performed in dynamic mode, using the amplitude as feedback channel for topographic determination (Supplementary Figures 13a and 13c). An additional phase locked loop (PLL) feedback was enabled, so changes in the oscillation phase are revealed by detecting the frequency shift required to keep the oscillation phase

constant (Supplementary Figure 13b). In KPM measurements, the retrace mode was employed, at a lift distance of ~12 nm, to record surface potential images (Supplementary Figure 13d) simultaneously with topography, using commercial NT-KP tips from Next-Tip S.L. Data has been processed using the WSxM software.[23]

Theoretical Section

General computational details

In order to shed light on the physical and electronic properties of the p-AP molecules integrated within the SAV created in the graphene we have carried out a battery of accurate Density Functional Theory (DFT)-based calculations by using an adequate combination of the localized-basis-set and plane-wave DFT-schemes as implemented in the FIREBALL[25] and QUANTUM ESPRESSO[10] simulation packages, respectively.

On one hand, we performed Density Functional Theory (DFT) calculations using the tight-binding FIREBALL code[25] to carry out structural pre-optimization studies of the systems, temperature-dependent molecular dynamics (MD) calculations and the simulation of tunnelling currents. This code is based on an optimized spatially-confined pseudoatomic basis set, and uses simulated thermal vibrations enabling the system to explore a wider range of energetic configurations in order to avoid the local minima.[25] In the calculations we have used a basis set of optimized sp^3d^5 numerical atomic orbitals (NAOs)[26] for C and N, $sp^3s^*p^{3*}$ for O and s for H, with cut-off radii (in a.u.): s = 4.0, p = 4.5 and d = 5.4 (C); s = 3.6, p = 4.1 and d = 5.2 (N); $s = s^*$ = 3.4, $p = p^*$ = 3.8 (O); and s = 4.1 (H). In our calculations we have used the Local Density Approximation (LDA) functional[27] and the ion-electron interaction has been modelled by means of norm-conserving scalar-relativistic pseudo-potentials.[28] The Brillouin zone was sampled only in the Γ point. On the other hand, in the efficient plane-wave code QUANTUM ESPRESSO[10] all our calculations accounted for dispersion forces within the DFT+D approach.[29] We have used the revised version of the generalized gradient corrected approximation of Perdew, Burke, and Ernzerhof (rPBE) to account for the exchange-correlation effects (XC),[30] and an empirical efficient vdW R^{-6} correction to add dispersive forces to

conventional density functionals.[29,31] Within this atomistic simulation package the Kohn-Sham equations are solved using a periodic supercell geometry. Rabe-Rappe-Kaxiras-Joannopoulos (RRKJ) ultrasoft pseudopotentials[19,32] have been used to model the ion-electron interaction in the H, C, N and O atoms. To be fully consistent the Brillouin zone was again sampled only in the Γ point for all the systems analysed. The one-electron wave-functions are expanded in a basis of plane-waves with energy cut-offs of 400 and 500 eV for the kinetic energy and for the electronic density, respectively, which have been adjusted to achieve sufficient accuracy to guarantee a full convergence in total energy and density.

Bader analysis

This approach constitutes a very intuitive and clever way of dividing molecules and atomic systems into atoms. The definition of an atom within this formalism is based purely on the electronic charge density and uses what are called zero-flux surfaces to divide atoms. A zero-flux surface is a 2D surface on which the charge density is a minimum perpendicular to the surface. Typically in molecular systems, the charge density reaches a minimum between atoms and this is a natural place to separate atoms from each other. Besides being an intuitive scheme for visualizing atoms in molecules, Bader's definition is often useful for electronic charge analysis. For example, the charge enclosed within the Bader volume is a good approximation to the total electronic charge of an atom. The charge distribution can be used to determine multipole moments of interacting atoms or molecules. Bader's analysis has also been used to define the hardness of atoms, which can be used to quantify the cost of removing charge from an atom. We have computed the electronic charge located around each atom after forming the whole interfacial system within this Bader framework.[33]

Theoretical STM Imaging

Theoretical STM calculations have been also performed for the p-AP adsorption configurations considered in this study, and compared with the experimental evidence. In order to obtain accurate STM images and tunnelling currents, we used an efficient STM theoretical simulation technique that includes a detailed description of the electronic properties of both the tip and the sample. Using this

technique, based on a combination of a Keldysh Green's function formalism and local orbital DFT,[34-36] we split the system into sample and tip, where the sample here is the p-AP functionalized graphene system and the scanning tip-model will be described below. Precisely, one of the main advantages of this technique is the possibility of functionalizing the scanning tip, which can be treated in an independent way from the sample (see below). Within this approach, in the tunnelling regime at low temperature, the STM current is given elsewhere[34-36] and depends on V_s – the surface voltage –, $\boldsymbol{\rho}_{tt}$ and $\boldsymbol{\rho}_{ss}$ – the density of states (DOS) matrices in the local orbital basis associated with the tip and sample –, and on $\boldsymbol{T}_{ts}$ and $\boldsymbol{T}_{st}$ – the local orbital Hamiltonian matrices coupling tip and sample.[34-36]

Supplementary Note 8: Transition-state Barrier Determination

Transition states (TSs) have been investigated here within the climbing-image nudge elastic band (CI-NEB) approach[37-39] implemented in the QUANTUM ESPRESSO package,[10] where the initial, the final, and a sufficient amount of intermediate image-states (typically between 10 and 15) were free to fully relax. This CI-NEB method has several desirable advantages, including the following: i) it converges to a Minimum Energy Path (MEP), providing sufficient resolution in the discrete representation of the path, when enough images are included in the seeking process, ii) it only requires evaluation of the interaction energy and the first derivative of the energy with respect to coordinates, iii) the convergence to the MEP is decoupled from the discrete representation of the path, making the former robust and the latter flexible, and, finally, iv) the method is guaranteed to provide a continuous path even when multiple MEPs exist. Within this approach we have computed all possible reaction mechanisms when the p-AP approaches the vacancy either by the OH or NH_2 groups. We have calculated height of the barriers, ΔE at the transition states (TS) for the double (-NH_2) dehydrogenation process of the p-AF molecule towards the integration of the molecule into the graphene lattice, as well as for the case in which a p-AP molecule would approach

to the SAV via de –OH terminating group towards its simple surface-induced dehydrogenation.

Supplementary Note 9: Minimum Energy Paths for the Dehydrogenation and Incorporation of Hydroxyl and Amino Groups

In Supplementary Figure 7 we show the MEP of the p-aminophenol molecule when approaches the graphene SAV from the side of the OH terminating group of the molecule. When the molecule is close enough from the surface an O—H stretching phonon is activated (by effect of the temperature and the collision between the surface and molecule electronic clouds) producing the detachment of the H atom from the OH group, which undergoes to chemisorb on-top an undersaturated C atom forming the SAV. The dehydrogenated p-aminophenol molecule links the remaining terminating O atom to other undersaturated C atom forming the SAV. The final state shows the axis of the molecule forming an angle of around 60° with the surface plane. C atoms involved in the adsorption of the detached H atom and the dehydrogenated p-aminophenol molecule show a non-negligible buckling > 0.3 Å off the graphene plane. In bottom panel of Supplementary Figure 7a we show energy (in eV) vs. reaction coordinate along MEP (referred to IS). In this case, the dehydrogenation TS energy barrier results to be about 0.8 eV, which makes the process also viable from the experimental point of view.

In Supplementary Figure 7b we show the MEP of p-aminophenol molecule when approaches the graphene SAV from the side of the NH_2 terminating group of the molecule. When the molecule is close enough from the surface an N—H stretching phonon in the NH_2 group is activated (by effect of the temperature and the collision between the surface and molecule electronic clouds) producing the detachment of one of the H atoms from the NH_2 group, which undergoes to chemisorb on-top an undersaturated C atom forming the SAV. The final state shows the axis of the molecule forming an angle of around 70° with the surface plane. C atom involved in the adsorption of the detached H atom and the singly

dehydrogenated p-aminophenol molecule show a non-negligible buckling > 0.3 Å off the graphene plane. Afterwards, once the p-aminophenol molecule has lost its first hydrogen from the NH_2 terminating group, and the detached H atom is chemisorbed on-top an undersaturated C atom forming the SAV, a second dehydrogenation of the remaining NH terminating group is produced: an N—H stretching phonon in the NH remaining group is activated (by effect of the temperature and the collision between the surface and molecule electronic clouds) producing the detachment of the last H atom from the NH group, which again undergoes to chemisorb on-top other undersaturated C atom forming the SAV. In this case the doubly dehydrogenated p-aminophenol molecule incorporates the remaining terminating N atom within the graphene lattice bonded to the three undersaturated C atoms forming the SAV in a substitutive manner, in such a way that the N atom fill the SAV hole leaving complete the graphene lattice. The final state shows the axis of the molecule forming an angle of around 90° with the surface plane. C atoms involved in the chemisorption of the detached H atoms show a non-negligible buckling > 0.3 Å off the graphene plane. The N atoms, although perfectly incorporated into the graphene lattice, shows a buckling > 0.6 Å off the graphene plane. In bottom panel of Supplementary Figure 7b we show energy (in eV) vs. reaction coordinate along MEP (referred to IS). The result of the TS energy barriers reveals that this double dehydrogenation process of the intact p-AP molecule is also efficiently catalysed by the SAV on the surface, yielding values of 1.3 and 0.7 eV for the first and the subsequent second dehydrogenation, respectively, of the amino terminating group.

Interestingly, although both processes are kinetically viable, the net gain of energy in the dehydrogenation of the OH terminating group is significantly lower than in the mentioned double ($-NH_2$) dehydrogenation (around 1.5 eV below), besides the fact that the remaining dehydrogenated oxygen would not be able to perfectly integrate within the SAV into the graphene lattice, only getting attached to one (of three) of the C atoms of the SAV.

The values obtained for the different transition state barriers could be slightly reduced (better capturing the catalysis of the processes) in the case of considering in the calculations a more massive substrate under the graphene, which would

geometrically increase up the number of atoms involved. Unfortunately, given the large amount of atoms already considered for the very demanding CI-NEB calculations, an increasing of the number of atoms involved in the calculations would turn this task into computationally unfeasible.

Supplementary Note 10: Scanning STM Tip-model: H-sensitized W-tip

In this study we have assumed to simulate the scanning with a W-tip (in order to mimic the experimental scanning sessions carried out with, in principle, a clean W-tip) formed by 5 protruding atoms (one of them in the apex) attached to an extended W(100)-crystal (in order to properly capture the necessary, in our framework, electronic structure register of the massive W-crystal). A pictorial scheme of the tungsten-tip model used in the calculations is shown in the top panel of the Supplementary Figure 6. The 5 protruding W atoms mentioned in the main text correspond to: 4 W ad-atoms continuing in a natural way the symmetry of the scaffolding W(100)-crystal plus another W-atom located on-hollow the adjacent four protruding W atoms attached to the crystal. This strategy permits to construct a sharp tip-model to maximize the efficiency in the tip-sample orbital overlapping during the scanning within the Keldish-Green formalism for the electronic propagation. Additionally, it is important to remark that this tip-model has already provided very successful results as compared with experimental STM images carried out with W-tips.[40] On the other side, from our expertise, and although the exact geometry of a real scanning tip is impossible to elucidate, this model has provided the best comparison with the experimental evidence, where the most important scanning contribution to the theoretical STM images comes from the atom in the apex. In previous literature by some of us, we report on the effect of the tip geometry in the theoretical STM images.[41] The fact of using such a sharp model with just one terminating atom in the apex has demonstrated to maximize the tip-sample orbital overlapping during the scanning. For a given clean (not poisoned or functionalized) W-tip, using other less reactive and less sharp tip-

model does not affect significantly the spatial resolution of the theoretical STM imaging.

Nevertheless, in the present case, there exist enough experimental evidences to think that the clean W-tip could have captured H atoms yielding a sort of H-sensitized W-tip, mainly due to the high spatial resolution exhibited by the experimental STM images (STHM). In an attempt to mimic such a remarkable experimental spatial resolution we have used, to simulate the theoretical STM images, a H-sensitized scanning W-tip constructed on the basis of the clean W-tip shown in the Supplementary Figure 6. This tip-model (shown in bottom panel of the Supplementary Figure 6) has been successfully used in previous literature to simulate STM images to mimic experiments carried out in chambers with a high saturation of atomic hydrogen,[41] yielding a remarkable enhancement of the theoretical spatial resolution as observed in the experiment. The tip-model is constructed by decorating with 5 H atoms our clean pyramidal W-tip. In order to have a hydrogen atom as the apex of the scanning tip four H atoms are located saturating the closest W—W bridges. This assumption is quite realistic given the high reactivity exhibited by these metal tips.[41]

Supplementary Note 11: Electric and Magneto Transport

At room temperature, after p-AP implantation, and from the slope of the R_{xy} vs. **H** plot at low fields (± 1 T) it is possible to obtain estimates of carrier (hole) densities in the range of 4-6×10^{13} cm^{-2} for samples on n-type substrates (1.5-1.8×10^{13} cm^{-2} for semi-insulator substrates). A similar study at low temperatures yields hole densities in the range 0.8-1.2×10^{13} cm^{-2} for samples on n-type substrates and no significant change with respect to room temperature for those grown on semi-insulator substrates. The presence of hole charge carriers has been signalled as a fingerprint of successful H intercalation[42] and it is noteworthy that this doping is retained in samples which have undergone the p-AP implantation procedure. In all cases, this Hall effect analysis would yield mobilities in excess of 10^3 cm V^{-1} s^{-1} but

we do not report more precise values since our geometrical factor is quite imprecise as our configuration deviates from Hall bar geometry. Supplementary Figure 10a shows the experimental raw data from these three channels for a p-AP implanted sample on an n-type SiC substrate at 1.8 K. The van der Pauw measurements R_{xx} and R_{yy} can be interpreted as the result of a combination of low and high field quantum effects (discussed below) together with a large positive magneto-resistance component, roughly quadratic in **H**, and a certain degree of asymmetry in **H** caused by the presence of classical Hall voltages. With a sample of macroscopic dimensions, it is not possible to conclude solely from electrical measurements as to the spatial dependence of these contributions. Most likely, the sample is not electrically homogeneous although it must contain a significant proportion of well-connected regions where single layer graphene properties are preserved.

The p-AP implantation protocol does not substantially alter the properties expected for a graphene system. Remarkably, p-AP implanted samples retain most of the electrical properties of a quasi-free standing graphene sample as it is shown in the resistance (*R*) vs. inverse temperature (*T*) measurements at zero magnetic field in Supplementary Figure 11 for a sample on semi-insulator SiC. Of paramount importance is the fact that the functionalized system has not a measurable gap at low temperatures (below 34 K); the thermally activated regimes at intermediate (between 34 and 140 K) and high temperature (above 140 K) regimes exhibit low activation energies.

Upon cooling below 34 K, *R* vs. **H** curves evidence the presence of Landau Levels ($E_{N,}$ labelled by quantum number *N*) in PAF implanted samples. Shubnikov-de Haas (SdH) oscillations are clearly shown in Supplementary Figures 10a and 10b for the three explored directions at 1.8 K. From a fit to $N = \mathbf{H}_0\ (1/\mathbf{H}) + g$ it is possible to obtain a slope $\mathbf{H}_0$ = (90.8 ± 0.04) T and a nearly zero intercept (g = (0.08 ± 0.1)) pointing to the appropriateness of describing these levels by those of single-layer graphene with massless Dirac´s fermions (Supplementary Figure 10c). At low fields, a weak localisation peak is also visible (inset to Supplementary Figure 10a). From the full-width at half-maximum of this peak (0.05 T from raw data in R_{xx}) it is possible to deduce a combined localisation and phase coherence length of 160 nm. Supplementary Figure 12a shows the temperature dependence of the amplitudes

of the SdH oscillations on a p-AP implanted sample on a semi-insulator substrate in the 2 to 34 K range. At 2K and 6 K, an N vs. $1/\mathbf{H}$ analysis yields $m_0\mathbf{H}_0$ = (75.4 ± 0.1) T and g = (0.02 ± 0.2). The anomalous behaviour of the amplitudes at 2 K (less intense than at 6 K) is not understood, but the amplitudes, $A(T)$, in the 6—34 K range are otherwise well described by the Lifshitz-Kosevich dependence $A(T) \approx u/\sinh(u)$, where $u = 2\pi^2 k_B T/\Delta E(H)$ and $\Delta E(H) = E_{N+1}(H) - E_N(H)$[43] (see Supplementary Figure 12b), allowing a determination of the Fermi energy of 520 meV from the slope $E_F/(2m_0\mathbf{H}_0)$ of a DE vs. $\mathbf{H}$ plot (see Supplementary Figure 12c) and from this and $\mathbf{H}_0$ a Fermi velocity of 1.7×10^6 m s^{-1}, from which an effective carrier mass of $0.01m_e$ results. The value of the Fermi energy determined from this analysis and the nature (holes) of the carriers determined from the magnetic field dependence of R_{xy} are in good agreement with those obtained in ARPES measurements.

Supplementary Note 12: AFM Characterization

Supplementary Figure 13 collects the results from AFM and KPM characterization of functionalized samples. Larger areas compared to STM analysis, can be measured with this technique, revealing the characteristic morphology of graphene grown on the Si-face of SiC, which is strongly dominated by SiC terraces as shown in Supplementary Figures 13a and 13c separated by step bunches, with heights ranging from 4 nm to 10 nm (height profile of Supplementary Figure 13e). The same morphology is observed for both, the pristine and the functionalized sample.

While the topography image (Supplementary Figures 13a and 13c) is strongly dominated by SiC terraces, additional information is extracted from simultaneously acquired frequency shift (see Supplementary Figure 13b) and surface potential images (see Supplementary Figure 13d). In both, a clear contrast is observed at the step bunches, which is a fingerprint of the presence of bilayer and trilayer graphene.[24,44] Three discrete potential values are observed in the corresponding histogram of the KPM image (Supplementary Figure 1f), where the low potential corresponds to areas of monolayer graphene and the higher

potential areas, at the step bunches, indicate the presence of bilayer and trilayer graphene. Small islands, ~200 nm of size, appear decorating the terraces and are assigned to bilayer islands from their contrast. The growth of these bilayer/trilayer patches along the terraces edges has been further assessed in reported studies with complementary techniques including KPM, Raman microscopy and low energy electron microscopy (LEEM),[45,46] and the contrast observed in surface potential is ascribed to different work functions values for different thickness, due to different substrate induced doping levels as a result of the different energy dispersions of mono- and bilayer graphene.[42,45,47] The measured surface potential difference between monolayer and bilayer graphene from Supplementary Figure 13d is 160 ± 60 mV. This value is similar for pristine and functionalized samples, indicating that the low p-AP density used in this study does not significantly alter the electrostatic properties of the graphene at these scales.

Higher magnification images show the presence of bumps with heights ranging from 6.5 to 9 Å high and around 25 Å wide. Our immobilized p-AP molecules are expected to be about 6.5 Å high, and therefore these bumps could correspond to the immobilized molecules enlarged by the tip-width.

Supplementary Note 13: Aminothiophenol Bond as a First-stage for Nanoparticle Immobilization

Supplementary Figure 14 shows the S *2p* Core level XPS spectrum after repeating our functionalization strategy using aminothiophenol molecules (see inset for the molecular description) instead of aminophenol. The red line corresponds to the fit of a single doublet component with a spin-orbit splitting of 1.2 eV. The maximum of the peak appears at a binding energy of 164.1 eV corresponding to the thiol group (SH) termination,[48] indicating that the linking of this molecule takes also place by dehydrogenation of the amino group, and exposing the –SH upwards. This indicates the generality of our experimental protocol, which on any amino-terminated molecule; and the opportunities to covalent link graphene to transition metal nanoparticles, preserving the properties of graphene.

Supplementary References

1. F. Speck, et al., The quasi-free-standing nature of graphene on H-saturated SiC(0001). Appl. Phys. Lett., 99, 122106 (2011).
2. Lv. Ruitao, et al., Nitrogen-doped graphene: Beyond single substitution and enhanced molecular sensing. Sci. Rep. 2, 1–8 (2012).
3. D. Yang, et al., Chemical analysis of graphene oxide films after heat and chemical treatments by X-ray photoelectron and Micro-Raman spectroscopy. Carbon 47, 145–152 (2009).
4. J. Choi, et al., Covalent functionalization of epitaxial graphene by azidotrimethylsilane. J. Phys. Chem. C 113, 9433–9435 (2009).
5. E. Mateo-Martí, et al., Self-assembled monolayers of peptide nucleic acids on gold surfaces: A spectroscopic study. Langmuir 21, 9510-9517 (2005).
6. A. Garcia-Lekue, Coordinated H-bonding between porphyrins on metal surfaces. J. Phys. Chem. C 116, 15378−15384 (2012).
7. M. Honda, et al., Electrochemical immobilization of biomolecules on gold surface modified with monolayered L-cysteine. Thin Solid Films 556, 307–310 (2014).
8. P. Merino, et al., Ortho and Para Hydrogen Dimers on G/SiC(0001): Combined STM and DFT Study. Langmuir 31, 233-239 (2015).
9. M. Telychko, et al., Achieving High-Quality Single-Atom Nitrogen Doping of Graphene/SiC(0001) by Ion Implantation and Subsequent Thermal Stabilization. ACS Nano. 8, 7318−7324 (2014).
10. P. Giannozzi, et al., QUANTUM ESPRESSO: A Modular and Open-source Software Project for Quantum Simulations of Materials. J. Phys.: Condens. Matter. 21, 395502 (2009).
11. S. García-Gil, A. Arnau and A. García-Lekue. Exploring large O *1s* and N *1s* core level shifts due to intermolecular hydrogen bond formation in organic molecules. Surf. Sci. 613, 102 (2013).
12. E. Pehlke, M. Scheffler. Evidence for site-sensitive screening of core holes at the Si and Ge (001) surface. Phys. Rev. Lett. 71, 2338 (1993).
13. A. Pasquarello, M. S. Hybertsen, G. M. Rignanese and R. Car. Core-level shifts in Si(001)-SiO_2 systems: The value of first-principle investigations.

Fundamental Aspects of Ultrathin Dielectrics on Si-Based Devices 47, 89 (1998).

14. J. Hand, T.-L. Chan, J.R. Chelikowsky. Quantum confinement, core level shifts, and dopant segregation in p-doped Si<110> nanowires. Phys. Rev. B 82, 153413 (2010).
15. B. Richter, H. Kuhlenbeck, H.-J. Freund and P.S. Bagus. Cluster core-level binding-energy shifts: The role of lattice strain. Phys. Rev. Lett. 93, 026805 (2004).
16. A. Catellani and A. Calzolari. Functionalization of SiC(110) surfaces via porphyrin adsorption: Ab initio results. J. Phys. Chem. C 116, 886 (2012).
17. E. Miniussi, et al. Non-local effects on oxygen-induced surface core level shifts of Re(0001). J. Phys. Chem. C 116, 23297 (2012).
18. N. Troullier and J. L. Martins. Efficient pseudopotentials for plane-wave calculations. Phys. Rev. B 43, 1993 (1990).
19. A. M. Rappe, K. M. Rabe, E. Kaxiras and J. D. Joannopoulos, Optimized Pseudopotentials. Phys. Rev. B. 41, 1227 (1990).
20. P. Merino, et al. Graphene etching on SiC grains as a path to interstellar polycyclic aromatic hydrocarbons formation. Nature Communications 5, 3054 (2014).
21. P. Feulner and D. Menzel. The Adsorption of Hydrogen on Ruthenium (001): Ad-sorption States, Dipole Moments and Kinetics of Adsorption and Desorption. Surf. Sci. 154, 465 (1985).
22. W. Strupinski, et al., Graphene Epitaxy by Chemical Vapor Deposition on SiC. Nano Lett. 11, 1786-1791 (2011).
23. I. Horcas, et al., WSXM: A Software for Scanning Probe Microscopy and a Tool for Nanotechnology. Rev. Sci. Instrument. 78, 013705 (2007).
24. O. Kazakova, et al., Epitaxial Graphene and Graphene–based Devices Studied by Electrical Scanning Probe Microscopy. Crystals 3, 191-233 (2013).
25. J. P. Lewis, et al., Advances and Applications in the FIREBALL Ab initio Tight-binding Molecular-dynamics Formalism. Phys. Stat. Sol. B. 248, 1989 (2011).
26. M. A. Basanta, et al., Optimized Atomic-like Orbitals for First-principles Tight-binding Molecular Dynamics. Comput. Mat. Sci. 39(4), 759-766 (2007).

27. P. Jelinek, et al., Multicenter Approach to the Exchange-correlation Interactions in Ab initio Tight-binding Methods. Phys. Rev. B. 71, 235101 (2005).

28. M. Fuchs and M. Scheffler, Ab initio Pseudopotentials for Electronic Structure Calculations of Poly-atomic Systems using Density Functional Theory. Comp. Phys. Comm. 119, 67-98 (1999).

29. S. Grimme, Semiempirical GGA-type Density Functional Constructed with a Long-range Dispersion Correction. J. Comp. Chem. 27, 1787 (2006).

30. J. P. Perdew, K. Burke and M. Ernzerhof, Generalized Gradient Approximation Made Simple. Phys. Rev. Lett. 77, 3865 (1996).

31. V. Barone, et al., Role and Effective Treatment of Dispersive Forces in Materials: Polyethylene and Graphite Crystals as Test Cases. Comput. Chem. 30, 934-939 (2009).

32. N. Mounet and N. Marzari, First-principles Determination of the Structural, Vibrational and Thermodynamic Properties of Diamond, Graphite, and Derivatives. Phys. Rev. B. 71, 205214 (2005).

33. R. F. W. Bader. In Atoms in molecules - A quantum theory (ed Bader, R. F. W.) Ch. 2, 13–49 (Oxford University Press, 1990).

34. J. M. Blanco, F. Flores and R. Pérez, STM-theory: Image Potential, Chemistry and Surface Relaxation. Prog. Surf. Sci. 81, 403-443 (2006).

35. J. M. Blanco, et al., First-principles Simulations of STM images: From Tunnelling to the Contact Regime. Phys. Rev. B. 70, 085405 (2004).

36. C. González, et al., Formation of Atom Wires on Vicinal Silicon. Phys. Rev. Lett.. 93, 126106 (2004).

37. Classical and Quantum Dynamics in Condensed Phase Simulations. B. J. Berne, G. Cicotti, D. F. Coker, Eds.; World Scientific Publishing Company: Singapore, 1998.

38. G. Henkelman and H. Jónsson, Improved Tangent Estimate in the Nudged Elastic Band Method for Finding Minimum Energy Paths and Saddle Points. J. Chem. Phys. 113, 9978 (2000).

39. G. Henkelman, B. P. Uberuaga, and H. Jónsson, A Climbing Image Nudged Elastic Band Method for Finding Saddle Points and Minimum Energy Paths. J. Chem. Phys. 113, 9901 (2000).

40. J. I. Martínez, et al., Simulating the Organic-molecule / Metal Interface TCNQ / Au(111). Phys. Stat. Sol. B. 248, 2044 (2011); J. I. Martínez, et al., Theoretical Characterization of the TTF / Au(111) Interface: STM-imaging, Band Alignment and Charging Energy. Org. Electr. 13, 399 (2012); C. Sánchez-Sánchez, et al., Chemistry and Temperature-assisted Dehydrogenation of $C_{60}H_{30}$ Molecules on TiO_2(110) Surfaces. Nanoscale. 5, 11058 (2013); A. L. Pinardi, et al., Sequential Formation of N-doped Nanohelicenes, Nanographenes and Nanodomes by Surface-assisted Chemical (Cyclo)dehydrogenation of Heteroaromatics. Chem. Comm. 50, 1555 (2013); A. J. Martínez-Galera, et al., Imaging Molecular Orbitals of PTCDA on Graphene on Pt(111): Electronic Structure by STM and First-Principles Calculations. J. Phys. Chem. C. 118, 12782 (2014); G. Otero-Irurueta, et al., On-surface Self-organization of a Robust Metal-organic Cluster based on Copper(I) with Chloride and Organosulphur Ligands. Chem. Comm. 51, 3243 (2015); J. I. Martínez, et al., Densely Packed Perylene Layers on the Rutile TiO_2(110)-(1×1) Surface. J. Phys. Chem. C. 119, 7809 (2015).

41. J. I. Martínez, et al., Improvement of Scanning Tunneling Microscopy Resolution with H-Sensitized Tips. Phys. Rev. Lett. 108, 246102 (2012).

42. C. Melios, et al., Carrier Type Inversion in Quasi-free Standing Graphene: Studies of Local Electronic and Structural Properties. Sci. Rep. 5, 10505 (2015).

43. Experimental Manifestation of Berry Phase in Graphene. A. F. Young, Y. Zhang and P. Kim. In H. Aoki, M.S. Dresselhaus (eds.), Physics of Graphene, NanoScience and Technology, Springer International Publishing, Switzerland (2014).

44. T. Filleter, et al., Local Work Function Measurements of Epitaxial Graphene. App. Phys. Lett. 93, 133117 (2008).

45. J. Eriksson, et al., The Influence of Substrate Morphology on Thickness Uniformity and Unintentional Doping of Epitaxial Graphene on SiC. Appl. Phys. Lett. 100, 241607 (2012).

46. C. E. Giusca, et al., Water Affinity to Epitaxial Graphene: The Impact of Layer Thickness. Adv. Mater. Interf. 2, 1500252 (2015).

47. D. Ziegler, et al., Variations in the Work Function of Doped Single- and Few-layer Graphene Assessed by Kelvin Probe Force Microscopy and Density Functional Theory. Phys.Rev B. 83, 235434 (2011).

48. David G. Castner, et al. X-ray Photoelectron Spectroscopy Sulfur *2p* Study of Organic Thiol and Disulfide Binding Interactions with Gold Surfaces. Langmuir 12, 5083-5086 (1996).
